# Application of High-Throughput Imaging Mass Cytometry Hyperion in Cancer Research

**DOI:** 10.3389/fimmu.2022.859414

**Published:** 2022-03-31

**Authors:** Marion Le Rochais, Patrice Hemon, Jacques-Olivier Pers, Arnaud Uguen

**Affiliations:** ^1^ B Lymphocytes, Autoimmunity and Immunotherapies, UMR1227, Immunology Department, Augustin Morvan Hospital, Brest, France; ^2^ Pathology Department, Augustin Morvan Hospital, Brest, France

**Keywords:** imaging mass cytometry, hyperion, cancer research, biomarker, tumoral microenvironment

## Abstract

Imaging mass cytometry (IMC) enables the *in situ* analysis of in-depth-phenotyped cells in their native microenvironment within the preserved architecture of a single tissue section. To date, it permits the simultaneous analysis of up to 50 different protein- markers targeted by metal-conjugated antibodies. The application of IMC in the field of cancer research may notably help 1) to define biomarkers of prognostic and theragnostic significance for current and future treatments against well-established and novel therapeutic targets and 2) to improve our understanding of cancer progression and its resistance mechanisms to immune system and how to overcome them. In the present article, we not only provide a literature review on the use of the IMC in cancer-dedicated studies but we also present the IMC method and discuss its advantages and limitations among methods dedicated to deciphering the complexity of cancer tissue.

## Introduction

Cancer is a leading cause of death worldwide in countries of all income levels. The number of cancer cases and deaths is expected to increase fast with the global growth and aging of the population (1). Therefore, cancer research remains a global issue in order to better understand its mechanisms and to discover new therapeutic solutions. Cancer progression is a multistep process requiring the participation of a multiplicity of heterogeneous components that can interact together (2). The tumor microenvironment (TME) and the interactions between tumor and non-tumor immune and non-immune cells are of crucial importance in the cancer initiation and progression, for example with the delivery of extracellular signals supporting tumor angiogenesis and promoting peripheral immune tolerance (3, 4). Direct clinical applications have emerged from the research on the TME as for examples prognostic applications based on tumor infiltrating lymphocytes (TILs) quantification and therapeutic (and theragnostic) applications with the immune checkpoint inhibitory anti-cancer immunotherapies as those targeting CTLA-4 and PD-1/PD-L1 axes in various solid cancers (5, 6).

To keep on investigating more comprehensively on TME’s components heterogeneity and distribution will allow a better understanding of the anti- and pro-tumoral mechanisms occurring within tumors and is a key to improve the management of patients with cancers towards better diagnostic, prognostic and theragnostic applications. In order to decipher the complexity of tumor tissue and TME, several methods have been developed to get a maximum of data from cell materials isolated from tumor tissue and, more recently, through *in situ* methods adding the spatial information of cells interactions within their tissue context to phenotypic data (7, 8).

Imaging mass cytometry (IMC) is one of these methods permitting the in-depth characterization of tissue until the single cell level and offering great possibilities of deciphering and correlating features of the tumor tissue and TME with biological and medical data. In this article, we first present this IMC method from a technical and practical point of view before providing a review on IMC-using cancer-dedicated studies and, finally, we discuss how IMC would integrate in current and future cancer research and clinical applications among other routinely-available to more innovative tissue study methods.

## The Imaging Mass Cytometry Hyperion^®^ Technology

### From Cytometry to Imaging Mass Cytometry

Cytometry and immunohistochemistry/immunofluorescence (IHC/IF) are routine methods daily used for diagnostic purpose to phenotype cells in liquid suspensions or within tissue samples respectively. Both of them use the highlighting of protein markers within cells and tissues thanks to antibodies (Abs) targeting epitopes of these protein markers. Abs are coupled with a revelation system permitting the detection and quantification of Abs-fixed within cells and tissue reflecting the abundance and localization of the targeted protein markers. Nevertheless, the number of markers co-analyzable at the same time using these diagnostic methods remains low because of the low number of revelation channels usable together that limits the number of differently-labeled Abs usable simultaneously. Indeed, standard fluorescence-based cytometry and IF methods are limited in their multiplexing possibilities by the overlapping-spectra of some fluorochromes rendering their proper signal indistinguishable for another and this limitation requires to use only a small number of fluorochromes with non-overlapping signals in these methods.

One way to increase the number of markers analyzable simultaneously was to use new revelation systems of the different Abs with no overlapping spectra and detection signal. Each metal isotope has a proper mass that allows its individual detection according to its time-of-flight (TOF) using mass spectrometry. So different metal isotopes can be used as revelation systems with TOF-based identification methods. The strategy using metal-tagged Abs and their detection using mass spectrometry has been first used for cytometry application through the development of the cytometry by TOF (CyTOF) mass spectrometry technique (9). Coupling several different metal isotopes to different Abs targeting several proteins and using mass spectrometry to detect them offers high multiplexing capacities in quantifying concomitantly different markers. The number of different metal isotopes available (about 50 available to date, in majority belonging to the lanthanide family) conditions the number of markers co-analyzable through this method (10). In this manner, the multiplexing capacity of CyTOF exceeds greatly the possibilities of fluorochrome–based cytometry.

**Figure 1:**
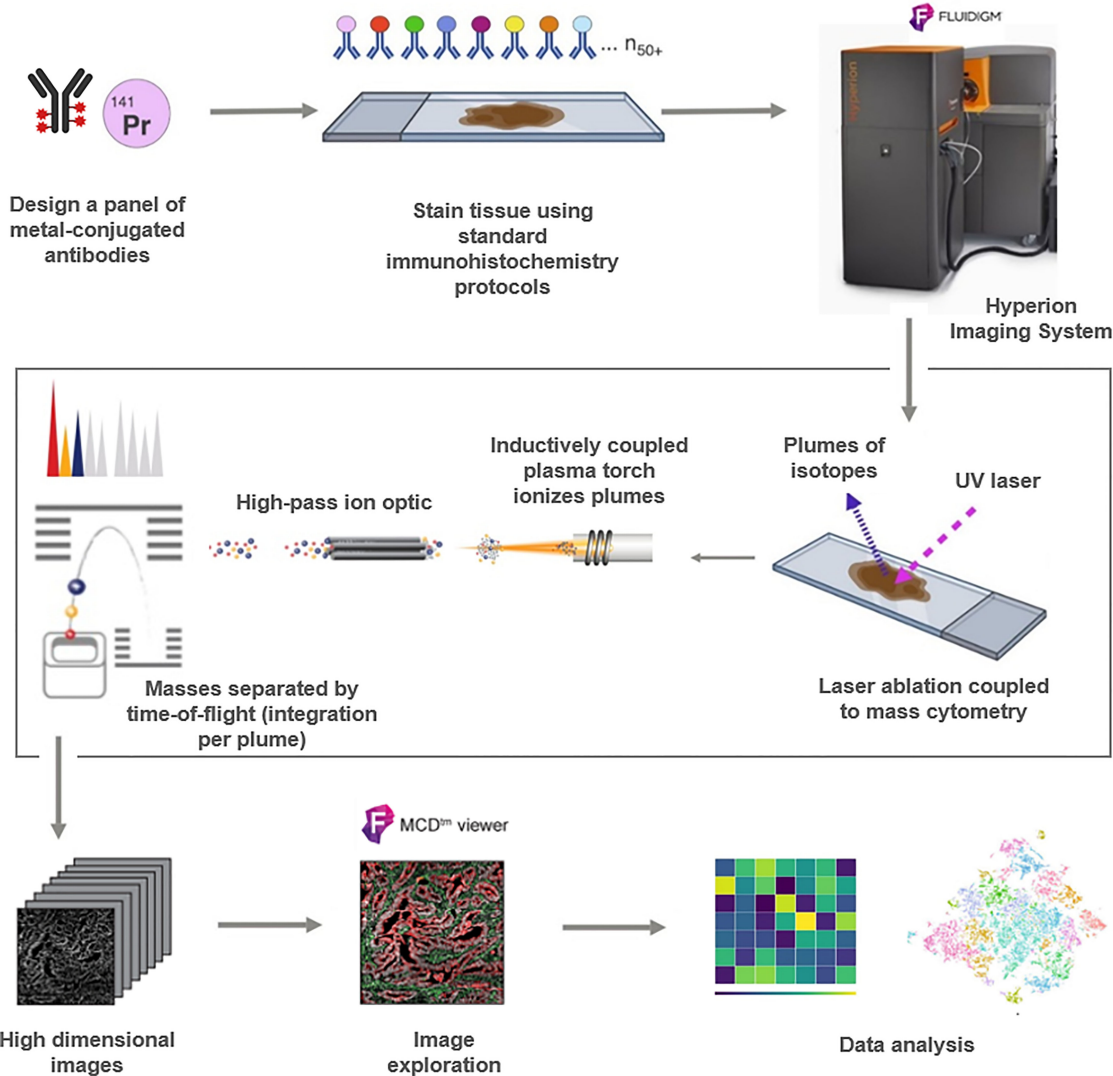
Schematic workflow of the Hyperion (Fluidigm) imaging mass cytometry (IMC) technique for protein profiling. Samples processing and design of the antibodies panel are followed by IMC staining and data acquisition in order to use the output data for analysis.

Coupling the CyTOF technology with laser ablation of cell-/tissue- material laid on glass slides, led to the imaging mass cytometry (IMC) method adding the tissue architectural information to CyTOF-based data. IMC has thus consisted in a major progress in the capacity of multiplexed immunodetection of several markers within tissue samples (9) with a capacity to evaluate up to 50 protein markers (with 50 metal-tagged antibodies) on each cell simultaneously (10) within the tissue, overcoming greatly the multiplexing possibilities of IHC/IF. Beyond the analysis of cells within their tissue context, the resolution of IMC (1µm² consisting in the cell/tissue surface ablated by the laser during each individual pulse) also permits to locate the proteins within the nuclear, cytoplasmic and membranous cell compartments. This IMC technology was commercialized in 2011 by the Fluidigm Corporation (South San Francisco, CA, USA) under the name Hyperion Imaging System permitting now the application of IMC analyses to every kind of samples laid on glass-slides as cells (11), Formalin-Fixed Paraffin-Embedded (FFPE) tissue sections (10) or snap-frozen tissues (2). IMC is thus particularly appropriate for the study of small and precious archived pathology samples as biopsies (12). As IHC/IF application, IMC processing follows the same steps of specimen pre-treatment prior the incubation of Abs and washing before the analysis of the Abs-signals within the cell/tissue specimens. Nevertheless, some specific concerns had to be taken into account in processing IMC analyses as mentioned hereafter. The main steps of IMC process are summarized in **Figure 1**.

### About Markers Panel Design and the Optimization of Immunolabeling

A major prerequisite in IMC application is the design of the Abs panel and the optimization of the incubation conditions of Abs on specimens. First, it is necessary to establish a list of markers whose study may help to solve a biological and/or clinical given question. Then, it is essential to get Abs targeting the different markers responding for each of them to the following criteria: 1) a storage condition allowing a metal-coupling (e.g. no albumin-containing storage medium) if the chosen Abs have not been previously coupled to metals, 2) a good staining obtained with an IHC validation step on a control tissue, 3) an antigen-retrieval pre-treatment condition during the IHC validation identical to those of the other Abs composing the panel [e.g. Tris-EDTA pH9 (13–16) or citrate pH6 (17)] keeping in mind that the different Abs will have to be co-incubated within a pool of Abs on the same pre-treated slide during IMC processing.

Of note, if IHC can give a trustable preview of the staining quality with the majority of Abs (17), issues can be particularly encountered with low-expressed markers that will merit to conjugate the corresponding Abs to the metal isotopes with the strongest detection index to optimize their further detection during IMC analysis.

To optimize the specific signal-to-noise ratio and to improve the detection of low-expressed markers, the incubation conditions can be also adapted depending on the Abs used to improve markers’ detection. For example, incubation/washing cycles can also be repeated with different Abs of a panel with only a little increase in the duration of the staining step but, overall, an optimization of the staining for low-expressed and highly expressed markers (18). Ijsselsteijn’s group compared incubations overnight at 4°C or for 5h at room temperature (17). Generally, low-expressed markers were more difficult to assess when their respective Abs were incubated at 4°C but they were more easily detected with incubation at room temperature. At the opposite, with a room-temperature incubation, Abs dedicated to high-expressed markers often led to more unspecific background signal that diminished with an incubation at 4°C.

Finally, in addition to the validation of each Ab separately using IHC prior any coupling to metal, it is also recommended to check the performance of metal-tagged Abs by performing an IMC analysis on a control tissue because metal-coupling can modify the affinity of the Abs for their epitope (2). Indeed, metal coupling can be performed thanks commercial kits permitting to break the disulfide bridges among the Abs to fix the metal within the Abs proteins molecules and this may modify the tridimensional structure of some Abs impairing their fixation to their targets.

After having validated the panel of Abs and optimized its incubation conditions, the IMC experiment can be performed with the Abs pooled at appropriate concentrations.

### From Slide to Imaging Mass Cytometry Image

If we consider a FFPE tissue sample to analyze using IMC, the FFPE tissue sections laid on glass slides have to be first deparaffinized. After that, slides are pretreated with an antigen retrieval buffer (pH6 or pH9 as determined and optimized above). An incubation period with a blocking buffer follows and tissue sections are then incubated with the mix of metal-tagged Abs in optimal time and temperature conditions. After incubation and washing, a DNA intercalating agent (for nuclei counterstaining) is incubated to end the immunolabeling step. The acquisition of Abs-signals for their subsequent analysis requires then the use of the Hyperion Imaging System.

The whole immunolabeled slide is then inserted into the ablation chamber of the Hyperion Imaging System. A first camera view allows for locating and selecting the region of interest (ROI) within the slide. This ROI can have been previously selected on the basis of a serial-stained tissue section or on the slide used for IMC itself providing the staining method prior to IMC do not impair the IMC process itself in terms of Abs fixation and mass spectrometry acquisition (2, 8, 19). Laser ablation is then performed on the ROI, 1µm² per 1µm². Each laser pulse ablates a tissue spot of 1µm², which is aerosolized, atomized, ionized, and carried by a helium gas to an argon flow with high time-fidelity into the inductively coupled plasma ion source for concomitant analysis by a TOF mass cytometer (CyTOF instrument). For each tissue spot, each isotope abundance can then be mapped back to the original coordinates. The tissue is analyzed spot-by-spot while the slide is moved under the laser for the scanning of the whole ROI (8, 10). The speed of the acquisition is about 100 minutes per 1 mm² of tissue. Each spot of ablated tissue corresponds finally to an image pixel associated with its content in different metal ions. The final result consists in a multichannel multiparametric image (reminding those obtained with fluorescence techniques) in form of a.mcd file gathering the data for the different pixels coordinates and their metal contains that will then allow the evaluation of the labeling intensity and the spatial distribution of the different Abs fixed in the tissue (9).

### From Imaging Mass Cytometry to Data Analysis

The analysis of the IMC multichannel image can be performed 1) from a morphological point of view as every microscopic multichannel fluorescence image thanks to viewers allowing the direct visualization of the.mcd generated image file assembling for each image pixel its coordinates and signal intensities for the different channels/metals isotopes/markers (e.g. MCD viewer developed by the manufacturer of the Hyperion automate Fluidigm), 2) from a bioinformatic point of view requiring dedicated pipelines. Bioinformatics pipelines for IMC analyses comprise notably image noise removal and cell segmentation which is a crucial key in the analytical process before exporting single cell data (i.e. coordinates in the image and multichannel signal intensities) for subsequent cell analysis. Cell analysis itself consists in cell phenotyping and quantifications through cytometry-inspired approaches based on the phenotypic characteristics of the cells (using non-supervised or supervised classification strategies) but also neighborhood analysis to point the spatial interactions between the cells within the tissue. Several free or commercial softwares and pipelines are available and keep on being developed to achieve the analysis of IMC data as proposed in the different studies summarized hereafter (14, 15, 20–22). Both the morphological approach and the bioinformatics analyses provide complementary data in the interpretation of IMC data.

In the field of cancer, IMC can be applied to different subtypes of tumors and samples to correlate IMC data with different biological or clinical questions as mentioned in the next section.

## Applications of Imaging Mass Cytometry in Cancer-Dedicated Studies

Pathology (histopathology and cytopathology) consist in analyzing cancer tissue and cell samples both for care and research purposes. It implies to analyze cell types, various tumor and non-tumor ones to provide qualitative and quantitative information correlated with medical and/or biological data. The morphological identification and analysis of cell-subtypes and -interactions is completed by additional qualitative and quantitative. Nevertheless, some limitations in these complementary studies of markers are linked notably to 1) the loss of detailed spatial tissue information in case of cells or cell-material extracted (e.g. DNA or RNA) from samples, 2) the limited capacity of repeating the analysis of single markers on samples with low cell/tissue amounts at risk of exhaustion, 3) the limited capacity of analyzing different markers simultaneously using IHC/IF methods and 4) the difficulty to get quantitative data in terms of signal intensity for a marker. IMC permits to overcome these limitations by 1) keeping the morphological information of cell/tissue material laid on glass slides, 2) requiring only a single cell/tissue slide, 3) allowing the co-analyze up to 50 markers simultaneously and 4) providing not only qualitative data but also quantitative information in terms of amount of metal-tagged Abs fixed in the corresponding spot of sample. For these reasons, IMC is particularly appropriate to analyze in-depth small amount/areas of cells/tissue cancer samples for correlation with medical and biological data as illustrated hereafter.

### Describing the Heterogeneity of Cancer Tissue, Cell-Subpopulations and Interactions

Investigating the heterogeneity of tumor tissues and cells is a frequent application of IMC used to report the detailed cell composition and cell interactions within a cancer tissue sample, or between different cancer samples per a same patient or between groups of patients (see **Table 1** for summary).

**Table 1:** Summary table of studies using IMC technology for the description of the cellular composition and their interaction in cancer tissue.

Cellular composition and interactions	Ref	Authors	Year	Main topics
Cellular heterogeneity				
	2	Elaldi et al. (2)	2021	Panel design and cellular phenotyping in cutaneous squamous cell carcinomas
	20	Xie et al. (20)	2021	TME variations in oral squamous cell carcinoma
	23	Aoki et al. (23)	2020	LAG3+ T cells (IL10+/TGFβ+) in classic-Hodgkin Lymphoma
	24	Li et al. (24)	2021	Proinflammatory CD3− CD4+ TNFa high Foxp3 high cells in lung squamous cell carcinoma
	25	Oetjen et al. (25)	2020	CD71+ CD235a+ Ki67+ erythroid cells in normal bone marrow samples and myelodysplastic syndromes
	26	Vassilevskaia et al. (26)	2018	PDL1 level in lung cancer
	27	Singh et al. (27)	2017	Large description of cell populations in colon cancer and Hodgkin lymphoma
	28	Tran et al. (28)	2020	p53 level in colorectal cancer
	29	Ijsselstein et al. (29)	2019	Panel design and cellular phenotyping in colorectal cancer
	30	Ravi et al. (30)	2021	Fine description of TME in glioblastoma (influenced by age)
	31	Gerdtsson et al. (31)	2018	Profiling ultra-rare circulating cells from a metastatic prostate cancer
	32	Batth et al. (32)	2020	Profiling ultra-rare circulating cells from osteosarcoma
	11	Bouzekri et al. (11)	2019	Phenotyping breast cancer cell lines
Novel phenotypes				
	25	Oetjen et al. (25)	2020	CD71+ CD235a+ Ki67+ erythroid cells in normal bone marrow samples and myelodysplastic syndromes
	33	Elyada et al. (33)	2019	CMHII + CD74+ CAFs in pancreatic ductal adenocarcinoma
	24	Li et al. (24)	2021	Proinflammatory CD3− CD4+ TNFa high Foxp3 high cells in lung squamous cell carcinoma
				
	23	Aoki et al. (23)	2020	LAG3+ T cells (IL10+/TGFβ+) in classic-Hodgkin Lymphoma
	34	Zhang et al. (34)	2019	EpCAM+ PD-L1+ CD4+ T cells in colorectal cancer
	35	Podojil et al. (35)	2020	B7-H4 + CD68 + cells as potential targets in urothelial carcinoma
Cellular interactions				
	36	Xiang et al. (36)	2020	Spatial interaction between CAFs and monocytic myeloid cells in lung squamous cell carcinoma
Cancer samples comparisons				
	37	Malihi et al. (37)	2018	EpCAM, PSA, and PSMA levels between primary and metastatic prostate cancer samples
	38	Cun et al. (38)	2021	Comparison of TME and prognosis between ovarian cancers
				
	39	Yusuf et al. (39)	2019	Comparison of the TME in non-small cell lung cancer between HIV+/HIV- patients

CAFs, Cancer-associated fibroblasts.

#### Deciphering the Complexity of Small Tissue Structures and Rare Cells

Among cancer-dedicated studies using IMC, several have focused mainly on reporting the feasibility of IMC analyses in various cancer subtypes and describing the composition of different cancer tissues as a technical preliminary step prior to future additional works investigating biological and/or clinical questions. These technical descriptive studies are particularly interesting as they contribute with other studies using IMC on reporting panels of Abs usable for IMC analyses (2, 20, 23–29). Beside quantifying cell-subpopulations, these studies have also pointed the feasibility of quantifying finely markers using IMC as PD-L1 expression in lung cancer and p53 in colorectal cancer samples (26, 28).

IMC particularly permits the in-depth study of microscopic tissue structure as tertiary lymphoid structures (TLS) in colorectal cancer or the interactions between tumor and immune cells within cancer tissue containing sometimes sparse tumor cells as Hodgkin lymphoma as report in the IMC study dedicated to these two cancers by Singh et al. who reported respectively Foxp3+ T cell-rich TLS in colorectal cancer and CD8+-T reg and Th2 cells circling tumor cells in Hodgkin lymphoma (27). Another illustration of fine deciphering the TME, Ravi et al. in-depth described glioblastomas tissues using IMC and they confirmed the major role of TME in the evolution of the tumors and their heterogeneity with a close correlation between the microenvironmental alterations, notably influenced by the age, and the selection of particular tumor subclones based on metabolic and genomic features (30).

Beside tissue-based applications, IMC has been also applied to cytology samples in which the high multiplexing capacities of IMC permits to deeply phenotype some rare cells as circulating tumors cells identified in blood samples of patients with cancers as prostate cancer by Gerdtsson et al. (31) or osteosarcoma by Batth et al. (32). Applications of IMC to deeply phenotype cancer cell lines have been also reported as the work on breast cancer cell lines by Bouzekri et al. (11).

#### Describing Complex and Novel Cell Phenotypes Within the Cancer Tissue

The combination of markers may permit the report of new cell populations as for example in the study by Oetjen et al. who described a new population of CD71+ CD235a+ erythroid cells with a high expression of the proliferative marker Ki-67 found within erythroid islands in normal bone marrow samples and myelodysplastic syndromes (25). Another example, Elyada et al. described, in pancreatic ductal adenocarcinoma, a novel population of cancer-associated fibroblasts (CAFs) that expressed MHC class II and CD74, but no classical co-stimulatory molecules and named it “antigen-presenting CAFs” (apCAFs). It was further demonstrate that this population is able to activate CD4+ T cells in an antigen-specific way in a model system, confirming its immune-modulatory ability (33). In lung squamous cell carcinoma, Li et al. also found a new subpopulation of CD3− CD4+ cells with high level of TNFa and Foxp3 which could modify the TME and play a proinflammatory role (24). Aoki et al. identified a special regulatory T cell–like subpopulation with an expression of lymphocyte activation gene 3 (LAG3+ T cells) in classic-Hodgkin Lymphoma, which was not find in normal reactive lymph nodes (23).Thanks to IMC, abnormal EpCAM+ PD-L1+ CD4+ T cells have been discovered in patients with colorectal cancer by Zhang et al. IMC also permitted to attest that these cells were also CCR5+ and CCR6+ and presented increased levels of phosphorylated p38 MAPK and MAPKAPK2 reflecting an activation of this signaling pathway (34). Studying urothelial carcinomas, Podojil et al. demonstrated using IMC the expression of B7-H4 on a subpopulation of CD68+ myeloid cells within the tumor that could consist in an alternative target in bladder cancers not responding to actual treatments (35).

#### Describing Cellular Interactions Within the Cancer Tissue

In addition to detect new cellular populations, IMC makes it possible to discover unsuspected spatial interactions between cells. In lung squamous cell carcinoma, Xiang et al. uncovered a notable spatial interaction between cancer-associated fibroblasts (CAFs) and monocytic myeloid cells in the TME and finally highlighted that the regulation of the recruitment and the differentiation of monocytes was coordinated by CAFs. They also demonstrated that the immunosuppressive microenvironment mediated by CAFs can be reversed by the inhibition of CCR2 and the elimination of ROS (36).

#### Comparing Different Cancer Samples, Histopathological and Clinical Subtypes

The comparison of different cancer samples in a same patient can help to better understand the mechanism implicated in its progression. As an example, IMC study of cells within prostatic and bone marrow samples in patients with metastatic prostatic adenocarcinomas has been performed by Malihi et al., who not only confirmed the maintained luminal prostate epithelial cell lineage of tumor cells between samples through the co-expression of EpCAM, PSA and PMSA, but also demonstrated that the level of expression of androgen receptor was higher in metastatic tumor cells than in prostatic ones (37).

The comparison of different subtypes of cancer affecting the same organ but known to have different prognosis and response to treatment can also be achieved using IMC analyses. For example, the TME of clear cell ovarian carcinoma has been described to be different from the one of high grade serous ovarian tumors by Cun et al. with notably features associated with poor prognosis (less intratumoral CD8+ T cells, higher density B7-H4^high^ Ki67^high^ cancer cells, and higher density of CD73^high^ cells) (38).

At the opposite, comparing identical subtypes of cancers in patients with different clinical conditions is also a potential question that could be addressed to IMC analysis as the study by Yusuf et al. comparing TME of non-small cell lung cancers in patients with and without HIV infection and pointing difference in terms of more pronounced PD-L1 expression by CD68 cells in HIV+ patients among others differences about MHC class I and II and proliferation markers (39).

### Correlating IMC Data With Patient’s Prognosis and Treatment Responses

Correlating IMC data with clinical and biological data about cancer evolution and response to anti-cancer treatment could not only improve the understanding of the different cancer diseases but could also point some new biomarkers helping to predict the evolution of cancer and its response to treatments. The studies using IMC on human samples in correlations with these clinical data are summarized in **Table 2**.

**Table 2:** Summary table of studies on patients’ prognosis and treatment responses using IMC technology in human and mouse cancerous tissues.

Prognosis and treatment responses	Ref	Authors	Year	Results
**Response to ICI treatments**	16	Hoch et al. (16)	2021	Chemokine landscape and immune infiltration characterization in metastatic melanoma sample
	40	Martinez-Morilla et al. (40)	2021	ICI potential biomarkers identification in metastatic melanoma
	41	Sanmamed et al. (41)	2021	Treatment resistance in non–small cell lung cancer
	42	Noac’h et al. (42)	2020	Patients’ outcomes in small-cell lung cancer
	43	Bortolomeazzi et al (43)	2020	Responses to anti-PDL1 agents in colorectal cancer
	44	Umemoto et al. (44)	2020	Comparison of the TME in early- to late-stage biliary tract cancer
	45	Zhu et al. (45)	2019	Immune biomarkers in pre- and on-treatment ICI in recurrent platinum-resistant epithelial ovarian cancer
	46	Zhang et al. (46)	2021	TME changes induced by neoadjuvant therapy in hepatocellular carcinoma
**IMC combination**	9	Giesen et al. (9)	2014	IHC and immunocytochemistry coupled with IMC in breast cancer
	14	Ali et al. (14)	2020	Genomic assays coupled with IMC in breast cancer
	15	Schulz et al. (15)	2018	Simultaneous detection and quantification of proteins, protein phosphorylations and transcripts in breast cancer
	19	Schulz et al. (19)	2021	Multispectral immunofluorescence coupled with IMC and omics data
	47	Kuett et al. (47)	2022	3D IMC in breast cancer
**Response to non-ICI treatments**	48	Carvajal-Hausdorf et al. (48)	2019	Cytotoxic T-cells improve effect of Trastuzumab in HER2+ breast cancer
	49	Hav et al. (49)	2019	TME characterization according to patients’ outcomes in diffuse large B cell lymphoma
	50	Colombo et al. (50)	2021	PD-L1/PD-1 levels in refractory and complete responders in diffuse large B cell lymphoma
	51	Hav et al. (51)	2019	CD8 spatial network alone could predict overall survival in diffuse large B cell lymphoma
	22,52	Zhu et al. (22, 52)	2020-21	Cellular comparison of LTS and STS in ovarian cancer
	21	Strobl et al. (21)	2018	Tumor-stroma interactions affect outcomes in ovarian cancer
**Platinum-based treatment**	53,54	Cao et al. (53, 54)	2019	Platinum deposition after Oxaliplatin in gastrointestinal malignancies
**Mouse models**	55	Chang et al. (55)	2016	Distribution of cisplatin in pancreas cancer PDX mice model
	56	Dey et al. (56)	2020	IL4, IL13 in *KRAS*-mutated pancreatic cancer mouse cell line
	57	Peran et al. (57)	2021	CDH11 level of CAFs in human and mouse pancreatic cancer
	58	Raj et al. (58)	2019	Improve effect of CAR-Tcells in metastatic pancreatic ductal adenocarcinoma PDX mice model
	59	Rinkenbaugh et al. (59)	2020	Pathway activation in triple negative breast cancer PDX mice model
	60	Liu et al. (60)	2021	Minimally invasive therapeutics delivery approach of CD40/PDL1to improve clinical response in a murine model of advanced triple negative breast cancer
	61	Guo et al. (61)	2021	MNK1/2-eIF4E axis involvement in postpartum breast cancer mouse model
	62	Somasundaram et al. (62)	2021	Resistance to anti-PD1 agents in mouse melanoma model

TILs, Tumor-infiltrating lymphocytes; TME, Tumor microenvironment; LTS, long term survivors; STS, short term survivors; PDX, patient-derived xenograft.

#### Searching for Predictive Biomarkers in the Field of Anti-Cancer Immune Checkpoint Inhibition

In the era of rapidly growing applications of anti-cancer immune checkpoint inhibitory immunotherapies (ICI), several studies have used IMC trying to find tumor tissue features that could consist in potential biomarkers able to predict the responses of patients to ICIs.

The study by Hoch et al. applied IMC in metastatic melanoma samples to demonstrate that particular CXCL9 and CXCL10 chemokines expression co-localized with CXCL13 expressing-dysfunctional T cells-TCF7+ naïve-like T cells as well as TLS and B cells whose recruitment was associated with anti-tumor immunity and response to ICI (16). Still in the field of melanoma, Martinez-Morilla et al. investigated using IMC for the correlation between TME features and the survival of patients with metastatic melanoma treated by ICI and they pointed some potential biomarkers of potential predictive interest as the level of beta2-microglobulin (40).

Sanmamed et al. have used IMC to study TILs within non-small cell lung cancer samples and identified a new burned-out CD8+ TIL subset (Ebo) that was especially accumulated within the TME and was a highly proliferative, overactivated, and apoptotic dysfunctional CD8+ tumor-infiltrating subpopulation that produced low amount of INFγ, functionally distinct from exhausted T cells. The expansion of this Ebo TIL population in a PD-1/B7-H1-dependent manner was associated with resistance to ICI (41). In the idea to stratify patients who will benefit the most from the combination of chemotherapy with anti-PD-L1 immunotherapy in small-cell lung cancer, IMC analysis have been conducted in order to find a predictive biomarker by Le Noac’h et al. Results showed that higher density of CD4+, CD8+, and regulatory T cells in the TME was notably associated with a longer progression-free survival (PFS) as was higher expression of granzyme B (42).

Bortolomeazzi et al. used IMC in colorectal cancer samples and highlighted that hypermutated colorectal cancers with responses to anti-PD-1 ICI contained high levels of cytotoxic and proliferating CD8+PD-1+ T cells with high interactions with PD-L1+ antigen presenting macrophages (43).

Trying to better understand the lack of response of patients with advanced biliary tract cancers to anti-PD-1 ICI treatment, Umemoto et al. analyzed the TME of biliary tract cancers patients samples using IMC and found that PD-1+CD8+ T cells, known to be important for the response to anti-PD-1 therapy, were not only higher in early-stage than in late-stage cancers, but they were also more numerous in direct interaction with tumor cells whereas CD8+ T cells in the stroma, at distance of tumor cells, were mainly PD-1- (44).

Zhu et al. used IMC to compare the tumor and TME composition between pre-treatment and on-ICI treatment (anti-PD-L1 plus anti-CTLA4 combination) samples of platinum-resistant epithelial ovarian cancers and they described that on-treatment decrease in tumor cells was associated with an increased number of CD8+ T cells, the increased in CD8+ and FoxP3+ cells being more important in patients responding the best to ICI (45).

Zhang et al. applied IMC analyses to post-neoadjuvant treatment tumor samples of patients with hepatocellular carcinomas treated by the combination of cabozantinib (tyrosine kinase inhibitor) and nivolumab (anti-PD-1 ICI) to point that samples of responders contained TLS and higher abundance of several immune cells as CD4+ and CD8+ T cells but also higher interactions between the different immune cells. In responders, the tumor cells also interacted more with lymphoid cells whereas in non-responders, CD8+ cells interacted with CD163+ macrophages. The more important the distance between B or T cells and CD163+Arg1+ macrophages was, the more important the response to treatment was. At the opposite, interactions between CD163+ Ki-67+ PD-L1+ macrophages and lymphoid cells were associated with response to treatment (46).

#### Improving the Prediction of Cancer Progression and Cancer Response to Non-ICI Treatments

Given their great experience of IMC applied to breast cancer samples, Bodenmiller’s team has particularly used IMC for prognostic applications. In the article by Ali et al., they have pointed some new features of TME including cellular neighborhoods that could be of potential prognostic interest in this field. Of note, in their work, IMC was often combined with genomics methods to better decipher the complexity of breast cancer at different level and to correlate this complexity with prognostic features (9, 14, 15, 19, 47). Still in breast cancer patients, Carvajal-Hausdorf et al. used IMC to correlate the expression of HER2 protein at plasma membrane of tumor cell, the quantification of CD8+ T cells and their spatial interactions with HER2+ tumor cells, and the response to anti-HER2 trastuzumab therapy. They demonstrated that the expression of the extracellular domain of HER2 and its proximity of CD8+ T cells were associated with the response to treatment supporting the role of the immune system in the action of this anti-cancer targeted therapy (48).

Hav et al. used IMC to phenotype cells within diffuse large B-cell lymphoma samples and have reported on the high prognostic value of CCR4+Tim3+PD-L1+ tumor cells in addition to spatial interactions implicating CD8+ T cells that were highly correlated with the clinical response to chemotherapy (49, 50). In addition, they observed also that granzymeB+CD8+ cytotoxic T cells were frequently associated with complete response to chemotherapy as was the position of CD4+ T cells closed to blood vessels. At the opposite, markers of exhaustion (Tim3 and Lag3) were associated with refractory disease as was the location of CD4+ T cell further away from the blood vessels. By leading separate analysis in large B-cell lymphoma of germinal center B-cell-like and non-germinal center B-cell-like subtypes, in the latter, high level of regulatory T cells was also associated with refractory disease (51).

Zhu et al. investigated for the prognostic significance of the TME composition analyzed using IMC in high-grade serous ovarian cancer and pointed some features associated with a long term survival (high levels of granzymeB+ CD8+ T cells, CD11b+Vista+ cells and interactions between granzymeB+ CD8+ T cells and Vista-CD4+ T cells; low levels of CD196+, CD45RO+ and CD73+ cells and poor interactions between CD73+ cells and Vista-CD4+ T cells, macrophages and B-cells) (22, 52). Strobl et al. also published some preliminary results on ovarian cancer trying to model the spatial composition and evolution of ovarian cancers TME on the basis of IMC data. This modelling can be of potential interest for better understanding tumor development and response to chemotherapy (21).

#### Visualizing the Tissue Deposits of Platinum-Based Treatment

Because IMC is based on the detection of metals within tissue samples, it not only permits the detection of the metals tagging the Abs used for the detection of proteins but also of metals already present in the tissue samples as platinum which is contained in some chemotherapy drugs. In this manner, IMC has also been used to investigate the platinum levels in non-tumor tissue and tumor tissue in patients treated with platinum-based chemotherapy. Cao et al. have notably pointed that the level of platinum within gastric cancer tumor samples, with a strong binding to collagen fibers, was correlated with an improved pathological response (53). They also proved that in patients with colorectal cancers treated with platinum-based chemotherapy skin platinum deposits lasted for several years after the treatment and could contribute to explain the mechanism of platinum-related peripheral sensory neuropathy as a side-effect of the treatment (54).

### Deciphering Cancer Progression and Response to Treatments in Animal Models

In addition to clinical patients’ samples, IMC is also applicable to study animal models of cancers and patient-derived tumor cell lines and xenografts. The application of IMC to these models could permit the comparison of cells and tissues at different time points of the treatment, after different treatment conditions, and/or to demonstrate the specific effect of some pathways on the architecture of tumor tissue and the aggressiveness of cancer.

An example, as performed by their team in human samples, Chang et al. also investigated for the distribution of platinum-based chemotherapy in normal and tumor tissues of mice with patient-derived xenografts of pancreas cancer finding the same fixation of platinum to collagen fibers encountered in human samples within normal and tumor tissue (55).

Dey et al. also applied IMC to study patients-derived pancreatic ducal adenocarcinoma cells xenografts in mice to decipher the interactions between cancer cells and host cells and the role of oncogenic role of *KRAS* mutations in terms of paracrine signaling (IL4 and IL13) and metabolic reprogramming of cancer (56). Peran et al. also used pancreas cancer xenograft in CDH11 deficient and wild-type mice models to demonstrate using IMC that the inhibition of CDH11, expressed by cancer-associated fibroblasts, caused a reduction in tumor growth, increased the tumor response to gemcitabine and was implicated in immunosuppression and extracellular matrix deposits consisting the highly fibrotic stroma of pancreatic ductal adenocarcinoma (57). Still about pancreatic ductal adenocarcinoma, Raj et al. have also used IMC analysis of patient-derived xenograft models to study the therapeutic effects of switchable CAR-T cells targeting HER2 this therapeutic effect co-occurring with a strong increase of the proliferation of T cells and an increase production of Granzyme B (58).

Rinkenbaugh et al. applied IMC to patient-derived xenografts models of triple negative breast cancer for comparison of tissue heterogeneity before and after chemotherapy treatment pointing, in post-treatment samples. They found an increased activation of PI3K/mTOR pathway and localized MAPK signaling suggesting the emergence of particular signaling niches following chemotherapy treatment that could contribute to the chemoresistance of these cancers (59). Liu et al. also used IMC in the field of triple negative breast cancers but analyzing not patients-derived xenografts but tumors arising in a mouse model of advanced triple negative breast cancer in which they compared effects of different drug-delivery methods in tumor and non-tumor tissues in order to search for the best efficiency on tumor and a minimal of treatment-related toxicity (60). Analyzing breast cancer cells grafted in different mice model including phospho-eIF4E deficient ones, Guo et al. has pointed the role of MNK1/2-eIF4E axis in the metastatic spreading of post-partum breast cancers and they subsequently used IMC to confirm the presence of high-levels of phospho-eIF4E high-expressing tumor cells and CD8+ T cells with activated dysfunctional phenotype markers in samples of humans with this particular clinical presentation of aggressive breast cancer pointing in this manner a potential new therapeutic target in this field (61).

In the field of anti-PD-1 ICI treatments, Somasundaram et al. generated a humanized-mouse melanoma model to investigate the tissue effects of anti PD-1 ICI. IMC analysis revealed the recruitment of FoxP3+ T cells and mast cells colocalizing in some tumor regions that contained also reduced HLA-class I expression and CD8+GranzymeB+ cells and were in this manner consistent with the acquisition of resistance to anti-PD-1 ICI treatment. This resistance related to mast cells infiltration was rescued by CD117 inhibiting mast-cell depleting therapies that could pave a new way to improve the efficacy of anti-PD-1 ICI treatment using combinatory approaches (62).

## The Place of Imaging Mass Cytometry in Current and Future Cancer Research

With its high level of multiplexing capacity, IMC has already been adopted for several applications, particularly in cancer research as evocated above. Nevertheless, it suffers from several limitations.

### Long Time and High Cost of IMC Analyses

Indeed, the speed of data acquisition (about 100 minutes per 1 mm² of tissue) is already a well-established limitation of IMC limiting the possibility of acquiring at reasonable cost and time data about large to whole slide areas. This requires the restriction of the area to analyze to small selected ROIs. To optimize the number of ROI and/or different tissues per IMC slide, Tissue Micro Array (TMA) cores sections can be used. The cost of IMC analyses is also proportional to the number and volume of metal-tagged antibodies to co-incubate in a tissue section, respectively related to the number of markers to analyze and to the surface of the ROI/tissue section to study. In addition to the cost of the IMC automat itself, all these factors make that IMC remains an expensive method actually hard to apply for routine diagnostic applications that would imply the analysis of numerous and large tissue samples of patients, especially with cancers. Nevertheless, one could expect that a progressive more widespread use of IMC might be also accompanied by a decrease in costs and by the optimization of the time and workflow of the process. In addition to its application on classical two-dimensional thin tissue sections or cells laid on glass-slides, IMC-based imaging could even be expanded to the tridimensional analysis of tissues through mass tomography. This method enables the analysis of cellular content and interactions in the volume and depth of the tissue and its feasibility has been demonstrated on a breast cancer sample by the team of Bodenmiller et al. (47). Nevertheless, this increasing level of analysis also greatly increases the time of acquisition and the related-cost of the analysis that constitutes a limitation in its wider application to date.

### IMC Among Other Immunolabeling Methods

Other multiplexed immunolabeling methods can also be used for the same purpose as IMC with lower level of multiplexing as multiplex immunofluorescence (until about 10 markers), or similar level of multiplexing as co-detection by indexing or fluorescent immune-histo-polymerase chain reaction (e.g. CODEX technology) and multiplexed ion-beam imaging (MIBI) TOF (up to 50 markers to date). Highly multiplexed methods have almost similar constraints in terms of time and cost and, for fluorescence-based methods, autofluorescence and potential fluorescence signal fading are other limitations that have not to be taken into account for metal-isotopes-based methods (IMC and MIBI). In comparison with the newer MIBI technology, IMC has the drawback to consist in a method implying laser tissue ablation (and so destruction) and to have a resolution of 1 µm whereas MIBI is non-tissue-destructive and permits a resolution of 200 nm (63). Both IMC and MIBI also share the limitation in terms of markers co-analyzable in a same slide dictated by the number of different metal isotopes available for Abs coupling (10, 17, 64). In a general manner, in the landscape of immunolabeling methods, multiplexed or not, it is actually reasonable to consider than low level of multiplexing, low cost and rapid methods for large (whole slide) image production and clinical applicability go together whereas high level of multiplexing require to focus on small tissue ROIs and require high cost and time of image acquisition and subsequent data analyses actually restricted to research applications and not to medical routine care. In this manner, in our opinion, a technical ecosystem combining both these low-level (for validation and clinical applications) and high-level (for research purpose of new phenotypic and spatial parameters of potential clinical interest) multiplexed immunolabelling methods as IMC remains necessary and complementary for translational research in cancer.

### Towards Deeper Tissue Analysis Combining IMC and Spatial Transcriptomics

During the last decades, great progress has been achieved in the capacity of comprehensive analyzing the genome (DNA) and the transcriptome (RNA) of cells including tumor ones, in terms of extraction from a whole tissue/mix of cells but also to provide information at the single cell level. Until recently, this molecular information was disconnected of the morphological and architectural data from where the cells/nucleic acid had been extracted but, with the current development of new technologies consisting in spatial transcriptomics, it will become more and more attainable to get comprehensive data about the expression of the genome through the transcriptome by analyzing the RNA of cells within their tissue context. These revolutionary methods will permit to keep on increasing the level of multiplexed-to-comprehensive analysis of tissue areas (to date) to single cells (in a near future) from fresh but also fixed cell and tissue samples. Facing with this ongoing technical revolution of spatial transcriptomics, one can ask for the place of multiplexed immunolabeling methods as IMC (65). Given cost and time considerations as well as the, availability of the different methods but also their different targets (cell RNA for spatial transcriptomics and proteins within cell but also non-cell components of the tissue for immunolabeling methods), both spatial transcriptomics and multiplexed immunolabeling methods will probably permit to get and correlate complementary data in successive tissue section or probably in a single slide. This will permit to get in-depth characterization of cells phenotypes and functional features interacting within normal and pathological tissues including cancer ones for a better understanding of cancer progression and the discovery of novel data of diagnostic, prognostic and theragnostic significance to improve the management of patients.

## Conclusion

Given the complexity and heterogeneity of cancer physiopathology in a general manner, methods allowing the simultaneous acquisition of data about different markers and preserving the information of tissue architecture, cell subpopulations quantifications and interactions consist in a major technical progress to better understand the mechanisms sustaining cancer progression. IMC is such a method applicable in cell and tissue cancer samples as those studied daily by pathologists for diagnostic, prognostic and theragnostic purposes. Combining to the immunostaining and digital pathology revolutions and at a time when molecular pathology is being combined to spatial data reflecting the architecture of cancer tissue through the development of spatial transcriptomics, no doubt that deciphering the phenotypes, functions and interactions of cells within cancer tissues through multiplex immunolabeling methods as IMC will be a key of discovery and translational applications for diagnostic, prognostic and theragnostic purposes conditioning the management of patients with cancers for better therapeutic choices at the era of personalized therapies in cancer.

## Author Contributions

MLR carried out the literature review. MLR and AU wrote the manuscripts. All authors contributed to manuscript revisions. All authors agree to be accountable for the content of the work. All authors contributed to the article and approved the submitted version.

## Funding

LBAI was supported by the AgenceNationale de la Recherche under the “Investissementd’Avenir” program with the Reference ANR-11-LABX-0016-001 (Labex IGO). The authors would like to acknowledge the Cytometry Core Facility Hyperion (Brest, France) for their technical assistance, as well as the European FEDER grant program Progos RU 000950 as well as “La Region Bretagne” and “La Ligue contre le cancer” for support.

## Conflict of Interest

The authors declare that the research was conducted in the absence of any commercial or financial relationships that could be construed as a potential conflict of interest.

## Publisher’s Note

All claims expressed in this article are solely those of the authors and do not necessarily represent those of their affiliated organizations, or those of the publisher, the editors and the reviewers. Any product that may be evaluated in this article, or claim that may be made by its manufacturer, is not guaranteed or endorsed by the publisher.
